# Attitude Heading Reference System Using MEMS Inertial Sensors with Dual-Axis Rotation

**DOI:** 10.3390/s141018075

**Published:** 2014-09-29

**Authors:** Li Kang, Lingyun Ye, Kaichen Song, Yang Zhou

**Affiliations:** College of Biomedical Engineering & Instrument Science, Zhejiang University, Hangzhou 310027, China; E-Mails: kang.li@outlook.com (L.K.); kcsong@zju.edu.cn (K.S.); chouyoung@zju.edu.cn (Y.Z.)

**Keywords:** AHRS, attitude sensor, MEMS inertial sensor, error compensation

## Abstract

This paper proposes a low cost and small size attitude and heading reference system based on MEMS inertial sensors. A dual-axis rotation structure with a proper rotary scheme according to the design principles is applied in the system to compensate for the attitude and heading drift caused by the large gyroscope biases. An optimization algorithm is applied to compensate for the installation angle error between the body frame and the rotation table's frame. Simulations and experiments are carried out to evaluate the performance of the AHRS. The results show that the proper rotation could significantly reduce the attitude and heading drifts. Moreover, the new AHRS is not affected by magnetic interference. After the rotation, the attitude and heading are almost just oscillating in a range. The attitude error is about 3° and the heading error is less than 3° which are at least 5 times better than the non-rotation condition.

## 1. Introduction

Attitude heading reference systems (AHRSs) which provide in-motion horizontal attitude as pitch and roll, and vertical angle as heading, are widely used in Autonomous Underwater Vehicles (AUVs) [1,2]. In order to afford sufficiently high precision, an AHRS often requires an inertial measurement unit (IMU) with high accuracy optical gyroscopes and quartz flexible accelerometers. It is too expensive to the low-cost applications. Moreover, the size of the AHRS is large. It does not fit the small sized and low cost AUVs such as the followers of a multi AUVs cooperative navigation system [3]. The position of the followers could be obtained from the leaders, while the attitude and heading should be gathered by itself. A thrifty way of doing this is using a digital magnetic compass [4,5]. These devices are low-cost and could achieve a heading accuracy of several milliradians. However, the accuracy drops easily while encountering electromagnetic interference such as the motors and the solenoid for the emergency jettison in the AUVs. It is not robust in such a harsh environment. With the fast development of low-cost Micro-Electro-Mechanical System (MEMS), the AHRS with MEMS inertial sensors could be a potential method.

Nowadays, the bias instability of the commercial MEMS gyroscopes is about several or more than 10°/h [6,7]. It is not enough for an AHRS, but the output accuracy could be improved by the IMU rotation technology [8,9]. The IMU rotation technology was initiated by the North Atlantic Treaty Organization (NATO) in the 1980s for marine inertial navigation systems [10]. This technology includes two main types, the single-axis rotation type such as MK39 and WSN-7B [11] and the dual-axis rotation type such as MK49 and WSN-7A [12,13]. It could reduce part of the inertial sensors' drifts for the single-axis rotation type; while the drifts of all the inertial sensors could be removed for the dual-axis type. Recently, this technology is still developing. The rotation scheme would affect the compensation result. Yuan *et al.* proposed an 8-sequence and a 16-sequence rotation scheme for the dual-axis rotational INS, which could compensate not only the drifts but also the scale factor errors of the gyroscopes [10]. The mounting errors between the IMU's body frame and the rotation table's frame must be calibrated since these errors will significantly affect the output attitude. Song *et al.* introduced the thin-shell algorithm to alignment these errors [14].

In recent times, this technology has been extended to the MEMS inertial sensors fields. Iozan *et al.* designed a north finding system using a MEMS gyroscope with a single-axis rotary table to compensate the gyroscope's bias [15]. Wei *et al.* introduced a MEMS gyroscope north finding system for mobile robot with a single-axis turntable [16]. It could detect the heading angle while the robot is stationary. Renkoski proposed a dual-axis continues rotation MEMS IMU for gyrocompassing applications. With a baseball stitch like slew, the heading angle error was almost not increasing in 60 s, and the north finding accuracy is improved compared with the 2-postion method [17].

This paper introduces IMU rotation technology into MEMS inertial sensors to produce an AHRS. The AHRS is self-contained and could provide in-motion horizontal attitude and heading. The rest paper is organized as follows. Section 2 explains the problem in mathematics. Section 3 proposes the AHRS and its rotary scheme according to the design principles. Section 4 analyzes the error sources of the AHRS and demonstrates the error compensation scheme in mathematics. The accuracy of the AHRS is evaluated with simulation in Section 5 and experiments in Section 6. Conclusions are drawn in Section 7.

## 2. Problem Statement

The coordinate frames used here are defined as follows:

1. The *n* frame is the ideal local level navigation coordinate frame with east-north-up geodetic axes.
2. The *n*′ frame is the real local level navigation coordinate frame. There are some errors between *n* and *n*′ frame owing to the sensor errors.
3. The *b* frame is the MEMS inertial sensor's body coordinate frame.
4. The *e* frame is the Earth coordinate frame.
5. The *i* frame is the nonrotating inertial coordinate frame.
6. The *r* frame is the rotation table's frame.
7. The *d* frame is the vehicle's body coordinate frame.

The attitude and heading are updated using the common navigation equation as [18]

(1) $${\dot{C}}_{b}^{n}=C_{b}^{n}\omega_{nb}^{b}\times$$

where $C_{b}^{n}$ is the attitude Direct Cosine Matrix (DCM) from the *b* frame to the *n* frame and $\omega_{nb}^{b}$ is the angular rate of the IMU in the *b* frame with respect to the *n* frame described as

(2) $$\omega_{nb}^{b}=\omega_{ib}^{b}-C_{n}^{b}\left(\omega_{ie}^{n}+\omega_{en}^{n}\right)$$

$\omega_{ib}^{b}$ is the angular rate measured by the gyroscopes in the *b* frame, $\omega_{ie}^{n}$ is the angular rate of the Earth's rotation in the *n* frame, $\omega_{en}^{n}$ is the rotation angular rate of the *n* frame with respect to the *e* frame, the (·)× is the skew symmetric matrix form of a cross-product which satisfies ***p*** × ***q*** = (***p***×)***q***.

Owing to the gyroscopes' measurement errors, the true $C_{b}^{n}$ is unavailable. In most case, we could only obtain its approximation with some errors as $C_{b}^{n^{\prime }}$. The Euler angle errors between the *n* frame and the *n*′ frame are described as [19–21]

(3) $$\dot{\alpha }=C_{\omega }^{-1}\left[\left(I-C_{n}^{n^{\prime }}\right)\left(\omega_{ie}^{n}+\omega_{en}^{n}\right)+\delta \omega_{ie}^{n}+\delta \omega_{en}^{n}-C_{b}^{n}\left(\delta \omega_{ib}^{b}+\delta K\omega_{ib}^{b}\right)\right]$$

where ***α*** = [*α_x_ α_y_ α_z_*]^T^ is the Euler angle errors, and $C_{\omega }^{-1}$is a 3 × 3 matrix defined as

(4) $$C_{\omega }^{-1}=\left[\begin{array}{ccc} \cos\alpha_{y} & 0 & \sin\alpha_{y}\\ \sin\alpha_{y}\tan\alpha_{x} & 1 & -\cos\alpha_{y}\tan\alpha_{x}\\ -\sin\alpha_{y}/\cos\alpha_{x} & 0 & \cos\alpha_{y}/\cos\alpha_{x} \end{array}\right]$$

***I*** is a 3 × 3 identity matrix, and $C_{n}^{n^{\prime }}$ is the DCM from the *n* frame to the *n*′ frame, as

(5) $$C_{n}^{n^{\prime }}=C_{\alpha_{y}}C_{\alpha_{x}}C_{\alpha_{z}}$$

where

(6) $$C_{\alpha_{x}}=\left[\begin{array}{ccc} 1 & 0 & 0\\ 0 & \cos\alpha_{x} & \sin\alpha_{x}\\ 0 & -\sin\alpha_{x} & \cos\alpha_{x} \end{array}\right],C_{\alpha_{y}}=\left[\begin{array}{ccc} \cos\alpha_{y} & 0 & -\sin\alpha_{y}\\ 0 & 1 & 0\\ \sin\alpha_{y} & 0 & \cos\alpha_{x} \end{array}\right],C_{\alpha_{z}}=\left[\begin{array}{ccc} \cos\alpha_{z} & \sin\alpha_{z} & 0\\ -\sin\alpha_{z} & \cos\alpha_{z} & 0\\ 0 & 0 & 1 \end{array}\right]$$

$\delta \omega_{ie}^{n}$is the angular rate error of the Earth's rotation in the *n* frame caused by the latitude error, $\delta \omega_{en}^{n}$ is the angular rate error caused by the velocity errors on the Earth, $\delta \omega_{ib}^{b}$ is the biases of the gyroscopes, and δ***K*** is the scale factor errors and axis misalignment errors matrix of the gyroscopes.

For an accurate AHRS, the goal is to make every effort to let $C_{b}^{n^{\prime }}$ close to the true DCM $C_{b}^{n}$. In other words, let ***α*** close to zero.When ***α*** is close to zero, for Equation (3), the part $I-C_{n}^{n^{\prime }}$ is near zero. Because the AUVs would not sail in a very high speed (commonly several meters per second), the $\delta \omega_{en}^{n}$ part is only several percent °/h if we regard the velocity as zero. Also, the $\delta \omega_{ie}^{n}$ part is several percent °/h if the latitude is roughly known, while the MEMS gyroscopes' error is several or more than 10°/h. In order to let ***α*** close to zero, the key is to make every effort to reduce the effect caused by the gyroscopes' biases, scale factor errors, and the axis misalignment errors.

(7) $$\int C_{b}^{n}\left(\delta \omega_{\text{ib}}^{b}+\delta K\omega_{ib}^{b}\right)dt\to {\left[\begin{array}{ccc} 0 & 0 & 0 \end{array}\right]}^{T}$$

## 3. Design Principles

Equation (7) can be separated with two parts: $C_{b}^{n}\delta \omega_{ib}^{b}$ and $C_{b}^{n}\delta K\omega_{\text{ib}}^{b}$. For the first part, $C_{b}^{n}$ is frequently changing through two axis rotations. Therefore, if the sum of $C_{b}^{n}$ could reach zero, the first part could be compensated. While with the introduction of the additional rotations, $\omega_{ib}^{b}$ would be much larger. This means that the second part would be increased. So the design principles should be as follows:

1. Try to keep $\int C_{b}^{n}=0$.
2. The rotation should be performed in both directions to compensate the $C_{b}^{n}\delta K\omega_{ib}^{b}$ part.
3. The rotational speed should be fast to ensure that the gyroscopes' biases and scale factor errors are as unchanged in one compensation cycle.

**Figure 1.**
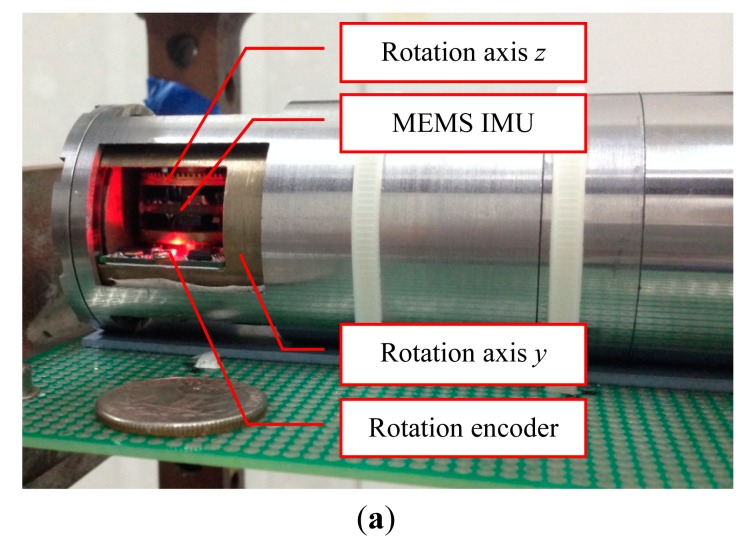

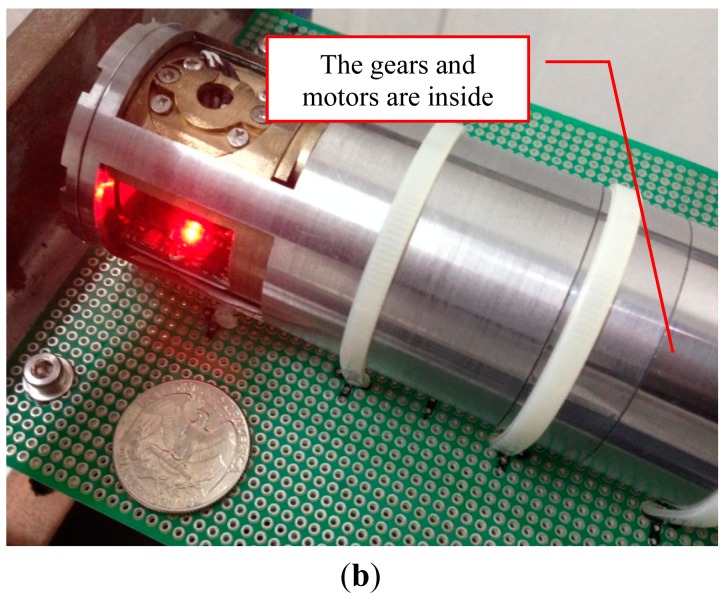
(**a**) Micro-Electro-Mechanical System (MEMS) inertial sensor based Attitude Heading Reference System (AHRS); (**b**) Size of the AHRS compared with a quarter dollar coin.

**Figure 2.**
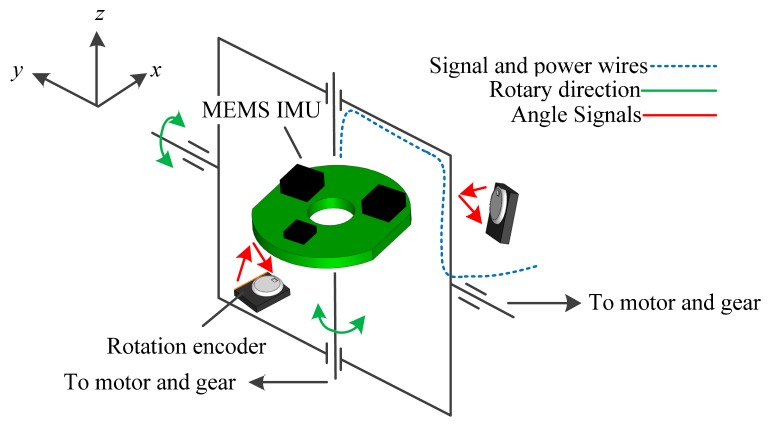
Block diagram of the AHRS.

In view of these principles, a MEMS inertial sensor based AHRS is designed as shown in Figure 1. The block diagram is shown in Figure 2.

**Figure 3.**
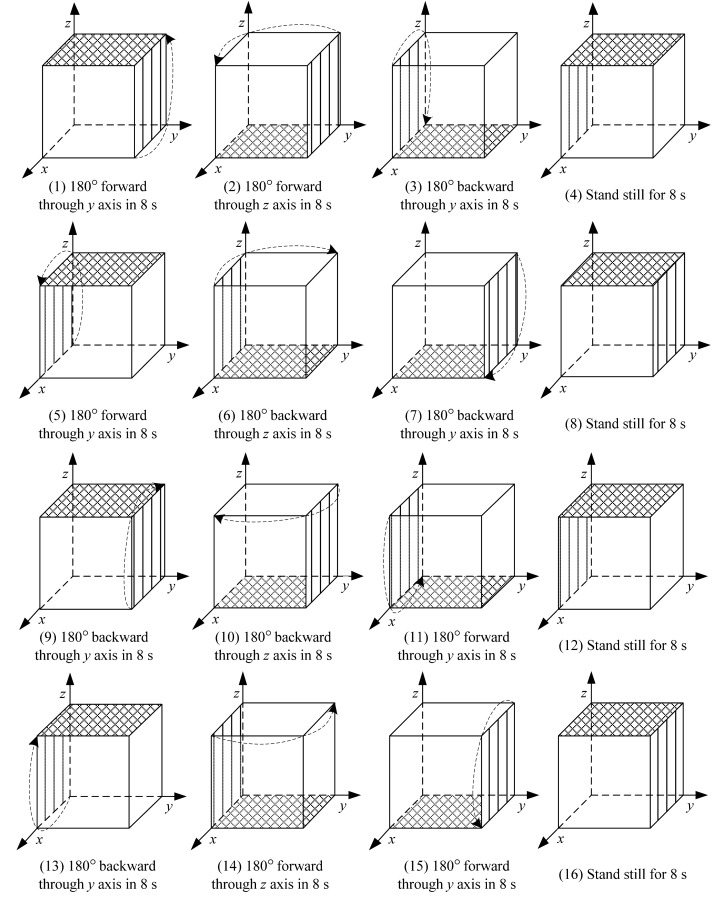
The rotary scheme. The directions of the rotations are according to the *d* frame.

The AHRS is designed as a *y-z* two axis reversible rotational structure. The rotation encoders are installed in both directions to measure the rotational angles since these extra angles should be compensated while outputting the attitude and heading. The signal and power wires are through the rotary centers to avoid the length changes. According to the rotational structure, a proper rotary scheme is considered, as presented in Figure 3.

Firstly, the rotation is performed in back and forth mode to avoid the influence of scale factor errors and axis misalignment errors and to keep the wires untwined. Secondly, since there are acceleration and deceleration stages for any rotation, the rotation is divided into 180° for one step. Then the duration of each direction and its opposite direction could be the same. Thirdly, 8 s is the fastest time that the motor and gear could finish the 180° turn.

## 4. Error Analysis and Compensation for the AHRS

In this section, we will have a delicate numerical analysis of the AHRS's attitude and heading errors according to the rotary scheme designed in Section 3. Besides, a new compensation algorithm to deal with the installation errors between the *b* frame and the *r* frame is proposed.

### 4.1. Installation Error Compensation

For a general AHRS, the outputs are the IMU's attitude and heading. While for a rotational AHRS, the additional information caused by the rotation should be compensated. The attitude and heading should be included in the DCM between the *d* frame and the *n* frame as

(8) $$C_{d}^{n}=C_{b}^{n}C_{r}^{b}C_{r_{0}}^{r}C_{d}^{r_{0}}$$

where *r*_0_ is the rotational table's frame at the initial time, and $C_{r_{0}}^{r}$ can be calculated through the rotation encoders. The AHRS could be initialized to let the *d* frame be parallel with the *r*_0_ frame by the adjustment based on the rotation encoders. Then, $C_{d}^{r_{0}}$ could be an identity matrix. When the IMU is fixed on the rotational table, it is hoped that the *b* frame is parallel with the *r* frame. However, there are always some installation errors between these two frames, which should be compensated.

**Figure 4.**
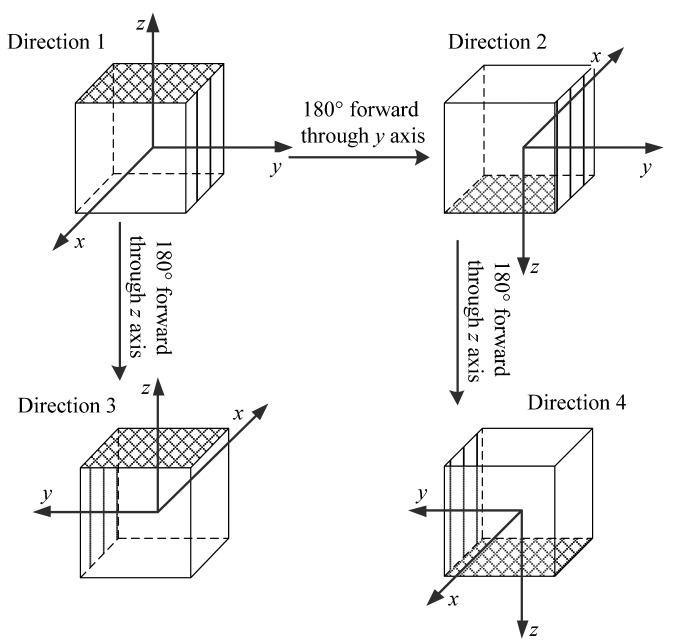
Rotation directions for installation error compensation.

Here, an optimization method is presented to detect the installation error DCM $C_{r}^{b}$. The rotary structure is rotated into four directions, displayed in Figure 4. The specific force of the IMU is collected as $f_{m}^{b}$, *m* = 1,2,3,4. There exists a relationship between ***f*** *^b^* and ***f*** *^r^* as

(9) $$T_{m}f^{r}=C_{b}^{r}f_{m}^{b}$$

where $f^{r}={\left[\begin{array}{ccc} f_{x}^{r} & f_{y}^{r} & f_{z}^{r} \end{array}\right]}^{T}$ is the specific force in the *r* frame which is unidentified, and the ***T****_m_* are 3 × 3 DCMs agreeing with the directions as

(10) $$\begin{array}{cc} T_{1}=\left[\begin{array}{ccc} 1 & 0 & 0\\ 0 & 1 & 0\\ 0 & 0 & 1 \end{array}\right] & T_{2}=\left[\begin{array}{ccc} -1 & 0 & 0\\ 0 & 1 & 0\\ 0 & 0 & -1 \end{array}\right]\\ T_{3}=\left[\begin{array}{ccc} -1 & 0 & 0\\ 0 & -1 & 0\\ 0 & 0 & 1 \end{array}\right] & T_{4}=\left[\begin{array}{ccc} 1 & 0 & 0\\ 0 & -1 & 0\\ 0 & 0 & -1 \end{array}\right] \end{array}$$

For the equation like Equation (9), if ***f*** *^r^* is known, $C_{b}^{r}$ can be uniquely solved by an optimization method using the singular value decomposition (SVD) as [22]

Step 1: Calculate the 3 × 3 matrix
  (11) $$H=\sum_{m=1}^{4}f_{m}^{b}{\left(T_{m}f^{r}\right)}^{T}$$
Step 2: Determine the SVD of ***H***
  (12) $$H={USV}^{T}$$
Step 3: Calculate
  (13) $$C_{b}^{r}={VU}^{T}$$

So the key is to identify ***f*** *^r^*. From Equations (9) and (10), we could get some interesting relations as

(14) $$\begin{array}{c} 4\left|f_{x}^{r}\right|=\left|\left[\left(T_{1}+T_{4})-(T_{2}+T_{3}\right)\right]f^{r}\right|=\left|C_{b}^{r}\left[\left(f_{1}^{b}+f_{4}^{b})-(f_{2}^{b}+f_{3}^{b}\right)\right]\right|\\ 4\left|f_{y}^{r}\right|=\left|\left[\left(T_{1}+T_{2})-(T_{3}+T_{4}\right)\right]f^{r}\right|=\left|C_{b}^{r}\left[\left(f_{1}^{b}+f_{2}^{b})-(f_{3}^{b}+f_{4}^{b}\right)\right]\right|\\ 4\left|f_{x}^{r}\right|=\left|\left[\left(T_{1}+T_{3})-(T_{2}+T_{4}\right)\right]f^{r}\right|=\left|C_{b}^{r}\left[\left(f_{1}^{b}+f_{3}^{b})-(f_{2}^{b}+f_{4}^{b}\right)\right]\right| \end{array}$$

where |·| is the operator to get the magnitude. $C_{b}^{r}$ is a DCM, it could change the direction of a vector and keep the magnitude unchanged. So Equation (14) can be simplified as

(15) $$\begin{array}{c} \left|f_{x}^{r}\right|=\left|\left(f_{1}^{b}+f_{4}^{b})-(f_{2}^{b}+f_{3}^{b}\right)\right|/4\\ \left|f_{y}^{r}\right|=\left|\left(f_{1}^{b}+f_{2}^{b})-(f_{3}^{b}+f_{4}^{b}\right)\right|/4\\ \left|f_{z}^{r}\right|=\left|\left(f_{1}^{b}+f_{3}^{b})-(f_{2}^{b}+f_{4}^{b}\right)\right|/4 \end{array}$$

And the signs of ***f****^r^* can be determined by making the *r* frame oblique in direction 1.

### 4.2. Attitude and Heading Errors Compensation

This part gives the numerical analysis of the attitude and heading errors based on the proposed rotary scheme to explain how this scheme can achieve the effect described in Equation (7).

There are acceleration and deceleration stages in each rotational step. The sinusoids are used here to simulate the rotational speed as

(16) $$\Omega =\frac{\pi }{2}\frac{\pi }{T}\sin\left(\frac{\pi }{T}t\right),t\in [0,T]$$

where *T* is the duration of one rotation step. Its integral form is

(17) $$\begin{array}{l} \Phi =\int_{0}^{t}\frac{\pi }{2}\frac{\pi }{T}\sin\left(\frac{\pi }{T}\tau \right)d\tau =\frac{\pi }{2}-\frac{\pi }{2}\cos\left(\frac{\pi }{T}t\right)\\ \Phi =\pi \text{when}t=T \end{array}$$

Then, the rotation steps from 1 to 16 can be expressed as

Step 1 (18) $$C_{r}^{r_{0}}=\left[\begin{array}{ccc} \cos\Phi & 0 & \sin\Phi \\ 0 & 1 & 0\\ -\sin\Phi & 0 & \cos\Phi \end{array}\right],\omega_{ib}^{r}=\left[\begin{array}{c} 0\\ \Omega \\ 0 \end{array}\right]$$
Step 2 (19) $$C_{r}^{r_{0}}=\left[\begin{array}{ccc} -\cos\Phi & -\sin\Phi & 0\\ -\sin\Phi & \cos\Phi & 0\\ 0 & 0 & -1 \end{array}\right],\omega_{ib}^{r}=\left[\begin{array}{c} 0\\ 0\\ -\Omega \end{array}\right]$$
Step 3 (20) $$C_{r}^{r_{0}}=\left[\begin{array}{ccc} \cos\Phi & 0 & \sin\Phi \\ 0 & -1 & 0\\ \sin\Phi & 0 & -\cos\Phi \end{array}\right],\omega_{ib}^{r}=\left[\begin{array}{c} 0\\ \Omega \\ 0 \end{array}\right]$$
Step 4 (21) $$C_{r}^{r_{0}}=\left[\begin{array}{ccc} -1 & 0 & 0\\ 0 & -1 & 0\\ 0 & 0 & 1 \end{array}\right],\omega_{ib}^{r}=\left[\begin{array}{c} 0\\ 0\\ 0 \end{array}\right]$$
Step 5 (22) $$C_{r}^{r_{0}}=\left[\begin{array}{ccc} -\cos\Phi & 0 & \sin\Phi \\ 0 & -1 & 0\\ \sin\Phi & 0 & \cos\Phi \end{array}\right],\omega_{ib}^{r}=\left[\begin{array}{c} 0\\ -\Omega \\ 0 \end{array}\right]$$
Step 6 (23) $$C_{r}^{r_{0}}=\left[\begin{array}{ccc} \cos\Phi & -\sin\Phi & 0\\ -\sin\Phi & -\cos\Phi & 0\\ 0 & 0 & -1 \end{array}\right],\omega_{ib}^{r}=\left[\begin{array}{c} 0\\ 0\\ \Omega \end{array}\right]$$
Step 7 (24) $$C_{r}^{r_{0}}=\left[\begin{array}{ccc} -\cos\Phi & 0 & \sin\Phi \\ 0 & 1 & 0\\ -\sin\Phi & 0 & -\cos\Phi \end{array}\right],\omega_{ib}^{r}=\left[\begin{array}{c} 0\\ -\Omega \\ 0 \end{array}\right]$$
Step 8 (25) $$C_{r}^{r_{0}}=\left[\begin{array}{ccc} 1 & 0 & 0\\ 0 & 1 & 0\\ 0 & 0 & 1 \end{array}\right],\omega_{ib}^{r}=\left[\begin{array}{c} 0\\ 0\\ 0 \end{array}\right]$$
Step 9 (26) $$C_{r}^{r_{0}}=\left[\begin{array}{ccc} \cos\Phi & 0 & -\sin\Phi \\ 0 & 1 & 0\\ \sin\Phi & 0 & \cos\Phi \end{array}\right],\omega_{ib}^{r}=\left[\begin{array}{c} 0\\ -\Omega \\ 0 \end{array}\right]$$
Step 10 (27) $$C_{r}^{r_{0}}=\left[\begin{array}{ccc} -\cos\Phi & \sin\Phi & 0\\ \sin\Phi & \cos\Phi & 0\\ 0 & 0 & -1 \end{array}\right],\omega_{ib}^{r}=\left[\begin{array}{c} 0\\ 0\\ \Omega \end{array}\right]$$
Step 11 (28) $$C_{r}^{r_{0}}=\left[\begin{array}{ccc} \cos\Phi & 0 & -\sin\Phi \\ 0 & -1 & 0\\ -\sin\Phi & 0 & -\cos\Phi \end{array}\right],\omega_{ib}^{r}=\left[\begin{array}{c} 0\\ -\Omega \\ 0 \end{array}\right]$$
Step 12 (29) $$C_{r}^{r_{0}}=\left[\begin{array}{ccc} -1 & 0 & 0\\ 0 & -1 & 0\\ 0 & 0 & 1 \end{array}\right],\omega_{ib}^{r}=\left[\begin{array}{c} 0\\ 0\\ 0 \end{array}\right]$$
Step 13 (30) $$C_{r}^{r_{0}}=\left[\begin{array}{ccc} -\cos\Phi & 0 & -\sin\Phi \\ 0 & -1 & 0\\ -\sin\Phi & 0 & \cos\Phi \end{array}\right],\omega_{ib}^{r}=\left[\begin{array}{c} 0\\ \Omega \\ 0 \end{array}\right]$$
Step 14 (31) $$C_{r}^{r_{0}}=\left[\begin{array}{ccc} \cos\Phi & \sin\Phi & 0\\ \sin\Phi & -\cos\Phi & 0\\ 0 & 0 & -1 \end{array}\right],\omega_{ib}^{r}=\left[\begin{array}{c} 0\\ 0\\ -\Omega \end{array}\right]$$
Step 15 (32) $$C_{r}^{r_{0}}=\left[\begin{array}{ccc} -\cos\Phi & 0 & -\sin\Phi \\ 0 & 1 & 0\\ \sin\Phi & 0 & -\cos\Phi \end{array}\right],\omega_{ib}^{r}=\left[\begin{array}{c} 0\\ \Omega \\ 0 \end{array}\right]$$
Step 16 (33) $$C_{r}^{r_{0}}=\left[\begin{array}{ccc} 1 & 0 & 0\\ 0 & 1 & 0\\ 0 & 0 & 1 \end{array}\right],\omega_{ib}^{r}=\left[\begin{array}{c} 0\\ 0\\ 0 \end{array}\right]$$

From Equation (8), $C_{b}^{n}$ can be conveyed as

(34) $$C_{b}^{n}=C_{d}^{n}C_{r_{0}}^{d}C_{r}^{r_{0}}C_{b}^{r}=C_{d}^{n}C_{r}^{r_{0}}C_{b}^{r}$$

And then the error compensation of the rotary scheme in each cycle can be obtained as follows

(35) $$\int_{0}^{16T}C_{b}^{n}\delta \omega_{ib}^{b}dt=\int_{0}^{16T}C_{d}^{n}C_{r}^{r_{0}}C_{b}^{r}\delta \omega_{ib}^{b}dt={\left[\begin{array}{ccc} 0 & 0 & 0 \end{array}\right]}^{T}$$

(36) $$\int_{0}^{16T}C_{b}^{n}\delta K\omega_{ib}^{b}dt=\int_{0}^{16T}C_{d}^{n}C_{r}^{r_{0}}C_{b}^{r}\delta KC_{r}^{b}\omega_{ib}^{b}dt={\left[\begin{array}{ccc} 0 & 0 & 0 \end{array}\right]}^{T}$$

Equations (35) and (36) show that the proposed rotary scheme could achieve the goal in Equation (7). The biases, scale factor errors, and axis misalignment errors can be completely counteracted.

## 5. Simulation

Three simulations are carried out to verify the rotation schemes. The conditions for the simulation are as follows: (1) the simulation's latitude is 30°; (2) the rotation time for each step is 8 s; (3) the simulation time is 3600 s.

**Figure 5.**
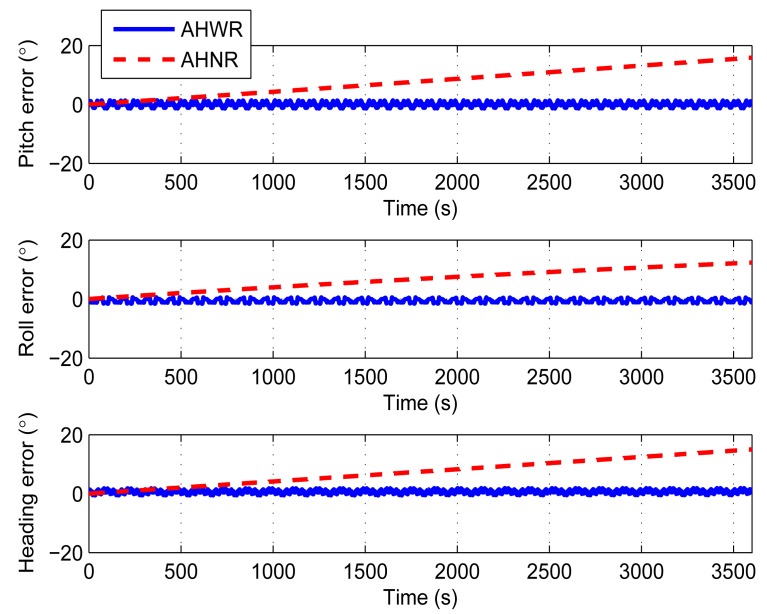
Attitude and heading errors comparison between attitude and heading sensing with rotations (AHWR) and attitude and heading sensing without rotations (AHNR) with constant gyroscope biases, scale factor errors, and axis misalignment angles.

The derivation in Sections 3 and 4 is based on the principle that the sensor errors are as unchanged in one compensation cycle. So, constant errors are applied in the first simulation to prove that the derivation is reasonable. The constant biases are set to 15°/h; the constant scale factor errors are set to 0.2% of the scale factor; and the constant axis misalignment angles are set to 0.6° for the simulation. The simulation also includes a comparison between two methods: (1) attitude and heading sensing with rotations (AHWR); (2) attitude and heading sensing without rotations (AHNR). Figure 5 and Table 1 show the comparison results. The attitude and heading errors are significantly decreased based on the rotation scheme. The errors do not increase with time compared with AHNR.

**Figure 6.**
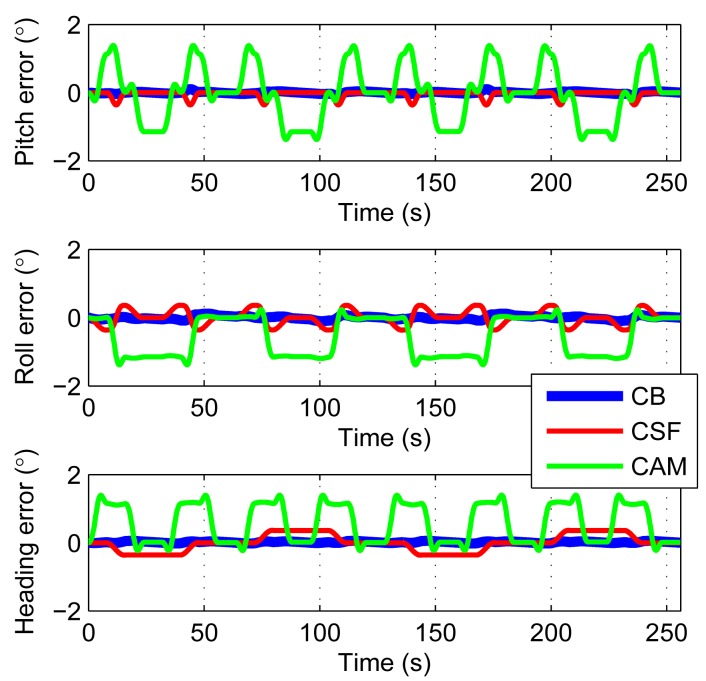
Attitude and heading errors caused by different error sources.

In order to separate the errors, the simulation also includes AHWR with constant gyro bias only (CB), AHWR with constant gyro scale factor errors only (CSF), and AHWR with constant axis misalignment angles only (CAM). Figure 6 shows the results within 2 compensation cycles. One compensation cycle lasts 128 s.The errors caused by gyroscope bias may increase in quarter of a cycle. So, it equals to 0.14° with gyroscope bias of 15°/h. The residual errors caused by the scale factor errors are about 0.4° and the remaining errors caused by the axis misalignment angles are about 1.5° when rotating 180°. They are all consistent with the error sources. Owing to the back and forth rotary scheme, the errors are only oscillating. Note also that the error levels of CSF and CAM are much larger than CB. So a potential way to improve the accuracy is to try to improve the techniques to produce MEMS gyroscopes with smaller scale factor errors and axis misalignment angles.

**Figure 7.**
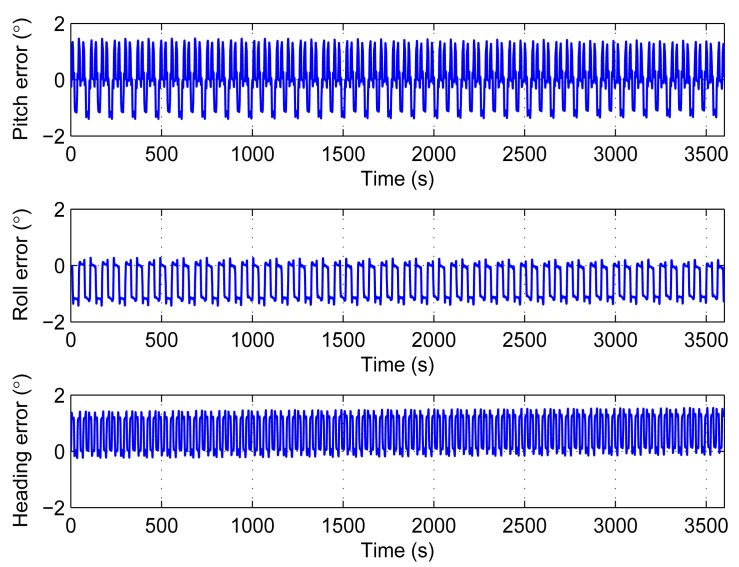
Average attitude and heading errors with long term drift added to bias. The dash lines are 1σ envelopes.

The compensation in Section 4.2 is based on the hypothesis that the biases and the scale factor errors are constant. Practically, for the real sensors, the errors should include not only constant part but also long term drift part and additional noise part. The second simulation adds random bias long term drift to the sensors. The rate is 15°/h, and the frequency is lower than 0.002 Hz. The simulation runs 100 times. The results are shown in Figure 7 and Table 2.

The results in Figure 7 are similar to Figure 5. It means that the long term drift can also be compensated by the rotations. The 1σ envelopes are within 0.04°. They are too close to the average values to see them clearly.

**Figure 8.**
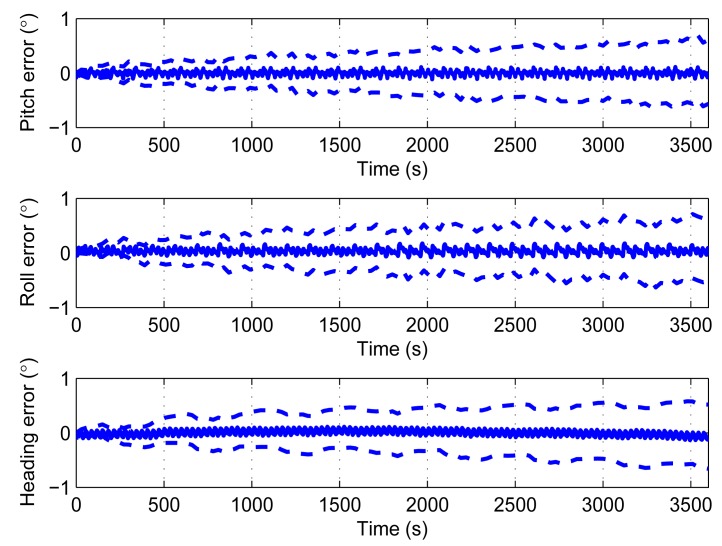
Average attitude and heading errors with noises added to bias and scale factor. The dash lines are 1σ envelopes.

The third simulation adds additional noises to the sensors. The rates are 0.009°/s/ $\sqrt{Hz}$ for bias, random data within ±0.25% for the scale factor. The simulation also runs 100 times. Figure 8 displays the average errors, as well as the respective 1σ envelopes.

In Figure 8, the average pitch error is within 0.15°, the average roll error is within 0.2°, and the average heading error is within 0.15°. The average errors are not growing with time. Although the average attitude and heading errors keep steady, its 1σ envelopes are increasing with time. After 3600 s, the errors reach 0.5° for both attitude and heading. So, with real sensors, the main error source is the random noises which cannot be compensated by rotation.

## 6. Experimental Results

Three experiments are carried out on the AHRS shown in Figure 1 to demonstrate the performance with real sensors. The parameters of the sensors are as follows:

1. Gyroscope: bias stability: around 15°/h, noise: 0.009°/s/ $\sqrt{Hz}$, axis misalignment: 0.6°;
2. Accelerometers: accuracy: 5 mg;
3. Rotation encoder: resolution: 0.03°.

The AHRS is fixed on an already calibrated turntable that the initial heading could be directly obtained from the turntable. The initial pitch and roll could be gathered from the accelerometers through two simple equations as [23]

(37) $$\theta_{\text{pitch}}=\arcsin\left(\frac{f_{y}^{b}}{g}\right)$$

(38) $$\theta_{\text{roll}}=\arcsin\left(\frac{-f_{x}^{b}}{g\cos\theta_{\text{pitch}}}\right)$$

where *g* is the gravity force.

**Figure 9.**
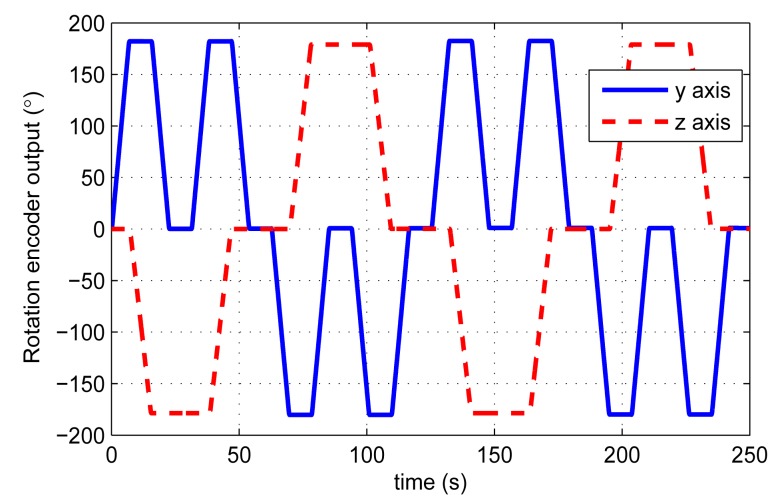
Rotation encoder outputs in two rotation cycles.

**Figure 10.**
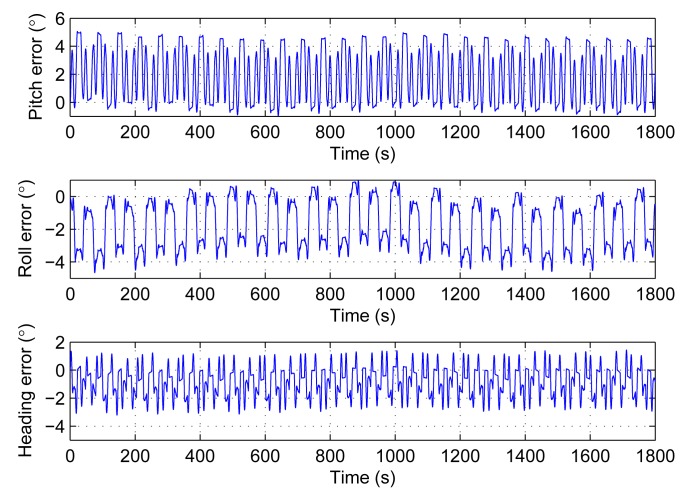
Attitude and heading errors of experiment 1 without installation error compensation.

**Figure 11.**
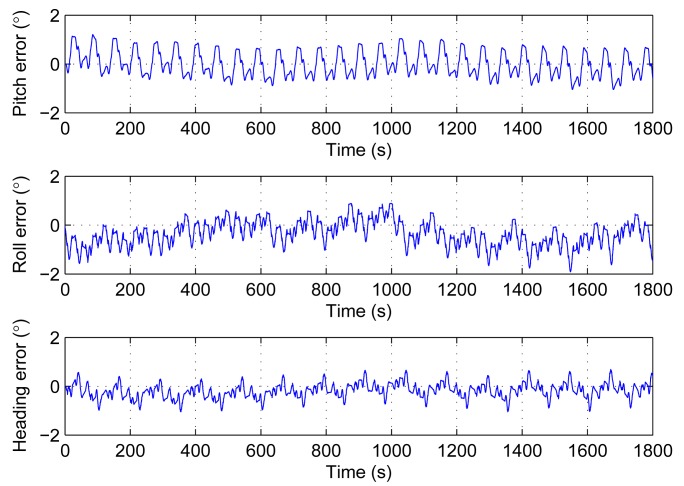
Attitude and heading errors of experiment 1 after installation error compensation.

The turntable keeps still in the first experiment. It means that the pitch, roll and heading are stable. The attitude and heading errors are the output drift. The first experiment is carried out three times and each one lasts 1800 s. The results are shown in Table 3. We take the first one as an example to give the experiment details. The results are performed from Figure 9, 10 and Figure 11.

Figure 9 displays the outputs of the rotation encoders within two rotation cycles. Owing to the rotation speed errors, one rotation cycle is not exactly 128 s.

Figure 10 gives the attitude and heading errors before the installation error compensation, while Figure 11 shows the results after the compensation. Both the errors in Figures 10 and 11 are oscillating within a range. However, it is clear that the range after the compensation is smaller. The statistics in Table 3 are based on Figure 11. The oscillatory ranges are all within 2°. According to the results of the simulations, these oscillations are caused by the scale factor errors and axis misalignment angles. Owing to the back and forth rotation mode, these errors would not increase. These results in Figure 11 are consistent with the simulations.

**Figure 12.**
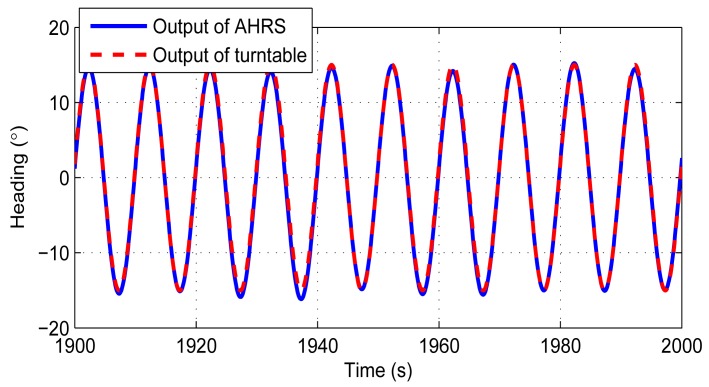
Heading comparison between the AHRS and the turntable.

**Figure 13.**
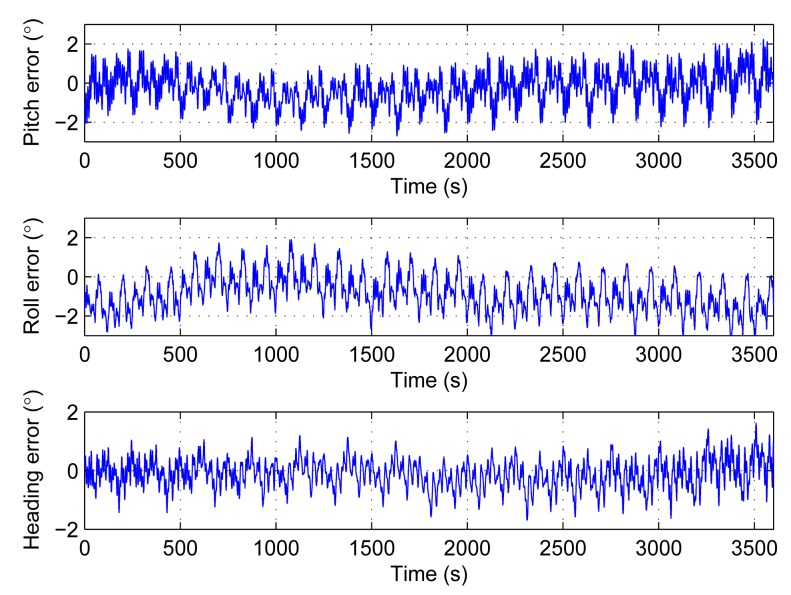
Attitude and heading errors of experiment 2.

In the second experiment, the turntable performed a ±15°, 0.1 Hz swing on the heading to evaluate the performance of the AHRS in dynamic environment. The reason we choose heading as the swing axis is that, for most of the AHRS, a roughly known pitch and roll can still be obtained from the accelerometers using the Equations (37) and (38) in motion situations, while the heading could only be obtained from the integration of the gyroscopes. The experiment is also performed three times, and each one lasts for 3600 s. The results are displayed in Table 4. We also take the first one as an example to give the experiment details. The results are performed in Figures 12 and 13.

The original output data rate of the turntable is 1 Hz. The data rate is increased by using a sinusoid wave curve fitting to 100 Hz. Figure 12 displays the heading outputs of the AHRS and the turntable within 10 cycles. The time delay between two outputs is about 0.14 s. These delays are already fixed in Figure 12. Figure 13 shows the errors of this experiment. The installation errors are already compensated in these two figures. The errors are a little bit larger compared with the stand-still condition. The pitch and roll errors are about 3°, and the heading error is within 2°.

**Figure 14.**
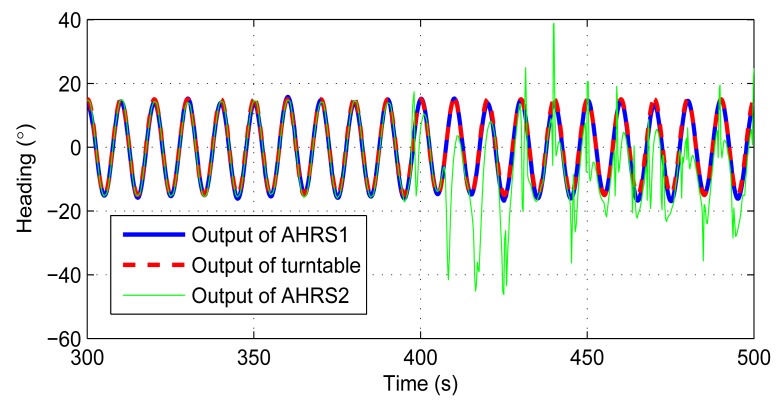
Heading comparison between AHRS1, AHRS2, and the turntable. Magnetic interference is added after 400 s.

**Figure 15.**
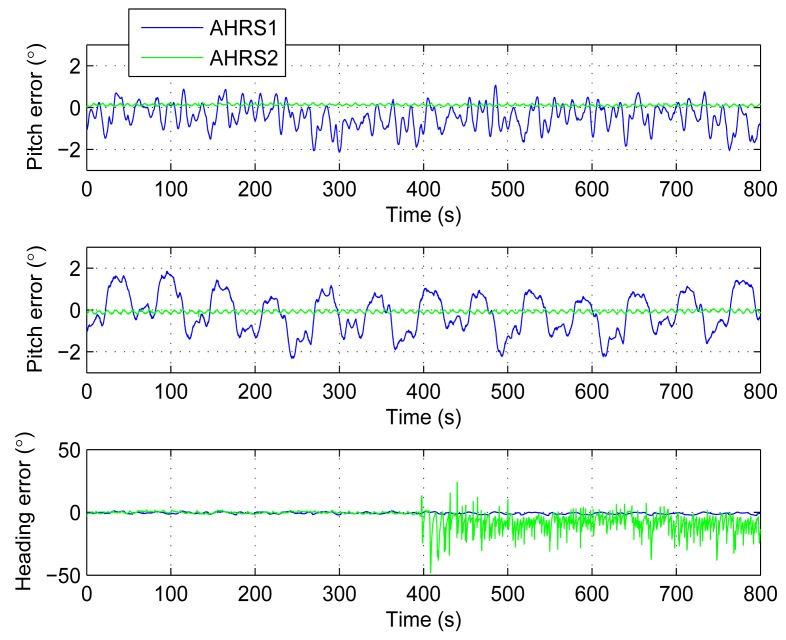
Attitude and heading errors comparison between AHRS1 and AHRS2. Magnetic interference is added after 400 s.

The third experiment is a comparison between the AHRS proposed by this paper (AHRS1) and a low cost digital magnetic compass based AHRS (AHRS2) to show that AHRS1 is more stable in some magnetic interference environment. The AHRS1 and AHRS2 are all put on the turntable. The turntable still performed a ±15°, 0.1 Hz swing on the heading. The experiment lasts for 800 s. For the first 400 s, we do not add any magnetic interference to these AHRSs. After 400 s, magnetic interference is added. The results are shown in Figures 14 and 15, and Table 5. In order to make waveforms clear, Figure 14 only shows the output between 300 s to 500 s.

For pitch and roll, the errors of AHRS2 are smaller than AHRS1, but the errors are on the same level. While for heading, the errors of AHRS1 and AHRS2 are on the same level before the magnetic interference is added. When the magnetic interference is added, the error statistics of AHRS1 keep small while the errors of AHRS2 are significantly increased.

## 7. Conclusions

A low cost and small size attitude and heading reference system based on MEMS inertial sensors is presented. The AHRS is based on a dual-axis rotation structure with a proper rotary scheme to compensate the attitude and heading drift caused by the large gyroscope biases, scale factor errors and axis misalignment errors. Also the installation angle error between the body frame and the rotation table's frame is calibrated through an optimization algorithm. Simulations and experiments are proposed to evaluate the performance of the AHRS. The attitude and heading drift are significantly reduced by the proper rotary scheme. The new AHRS is not affected by magnetic interference. The attitude error is about 3° and the heading error is less than 3°, which are at least 5 times better than the non-rotation condition. Furthermore, the errors are almost just oscillating within a range. Most of these oscillatory errors are caused by the scale factor errors and axis misalignment errors with additional rotations. So we believe that the accuracy could be further improved if the scale factor errors and axis misalignment errors of the MEMS gyroscopes could be reduced.

## Figures and Tables

**Table 1.** Statistics of Figure 5.

		**Mean**	**STD**	**Max**	**Min**
Pitch error (°)	AHWR	−0.039	0.758	1.406	−1.401
	AHNR	7.839	4.590	15.850	0

Roll error (°)	AHWR	−0.573	0.597	0.592	−1.603
	AHNR	6.626	3.568	12.360	0

Heading error (°)	AHWR	0.651	0.662	1.884	−0.603
	AHNR	7.458	4.323	15.001	0

**Table 2.** Statistics of Figure 7.

		**Mean**	**STD**	**Max**	**Min**
Pitch error (°)	Average of 100 times	0.002	0.787	1.462	−1.407
	1σ envelopes			0.031	

Roll error (°)	Average of 100 times	−0.574	0.573	0.299	−1.449
	1σ envelopes			0.026	

Heading error (°)	Average of 100 times	0.659	0.574	1.539	−0.244
	1σ envelopes			0.014	

**Table 3.** Statistics of the first experiment.

		**Mean**	**STD**	**Max**	**Min**
Pitch error (°)	1	0.015	0.514	1.209	−1.048
	2	0.707	0.952	2.837	−1.520
	3	−0.861	0.690	0.831	−2.780

Roll error (°)	1	−0.359	0.509	0.889	−1.909
	2	0.167	0.908	1.987	−2.245
	3	0.410	0.899	2.425	−1.227

Heading error (°)	1	−0.184	0.301	0.687	−1.050
	2	−0.264	0.603	1.055	−1.707
	3	0.290	0.884	2.414	−1.493

**Table 4.** Statistics of the second experiment.

		**Mean**	**STD**	**Max**	**Min**
Pitch error (°)	1	−0.248	0.871	2.223	−2.701
	2	−0.354	0.826	2.017	−2.939
	3	−0.255	0.743	2.205	−2.456

Roll error (°)	1	−0.835	0.908	1.888	−3.332
	2	0.009	1.124	2.989	−2.924
	3	−0.151	1.018	2.535	−2.935

Heading error (°)	1	−0.111	0.491	1.606	−1.685
	2	−0.137	0.732	1.751	−2.054
	3	−0.484	1.028	2.861	−2.867

**Table 5.** Statistics of the third experiment.

		**Mean**	**STD**	**Max**	**Min**
Pitch error (°)	AHRS1	−0.441	0.593	1.065	−2.127
	AHRS2	0.122	0.044	0.247	0.002

Roll error (°)	AHRS1	−0.149	0.935	1.843	−2.302
	AHRS2	−0.080	0.061	0.063	−0.318

Heading error (°) before interference added	AHRS1	−0.388	0.663	1.132	−2.069
	AHRS2	−0.146	0.755	1.855	−3.378

Heading error (°) after interference added	AHRS1	−0.919	0.709	0.751	−2.457
	AHRS2	−8.466	7.091	23.860	−48.260
